# Enhanced interlaminar excitation or reduced superficial layer inhibition in neocortex generates different spike-and-wave-like electrographic events in vitro

**DOI:** 10.1152/jn.00516.2017

**Published:** 2017-09-27

**Authors:** Stephen P. Hall, Roger D. Traub, Natalie E. Adams, Mark O. Cunningham, Ian Schofield, Alistair J. Jenkins, Miles A. Whittington

**Affiliations:** ^1^Hull York Medical School, University of York, Heslington, United Kingdom; ^2^Department of Physical Sciences, IBM Thomas J. Watson Research Center, Yorktown Heights, New York; ^3^Institute of Neuroscience, Newcastle University, Newcastle upon Tyne, United Kingdom; ^4^Department of Clinical Neurophysiology, Royal Victoria Infirmary, Newcastle upon Tyne, United Kingdom

**Keywords:** epilepsy, spike and wave event, synaptic excitation, synaptic inhibition, neocortex

## Abstract

Acute in vitro models have revealed a great deal of information about mechanisms underlying many types of epileptiform activity. However, few examples exist that shed light on spike-and-wave (SpW) patterns of pathological activity. SpW are seen in many epilepsy syndromes, both generalized and focal, and manifest across the entire age spectrum. They are heterogeneous in terms of their severity, symptom burden, and apparent anatomical origin (thalamic, neocortical, or both), but any relationship between this heterogeneity and underlying pathology remains elusive. In this study we demonstrate that physiological delta-frequency rhythms act as an effective substrate to permit modeling of SpW of cortical origin and may help to address this issue. For a starting point of delta activity, multiple subtypes of SpW could be modeled computationally and experimentally by either enhancing the magnitude of excitatory synaptic events ascending from neocortical layer 5 to layers 2/3 or selectively modifying superficial layer GABAergic inhibition. The former generated SpW containing multiple field spikes with long interspike intervals, whereas the latter generated SpW with short-interval multiple field spikes. Both types had different laminar origins and each disrupted interlaminar cortical dynamics in a different manner. A small number of examples of human recordings from patients with different diagnoses revealed SpW subtypes with the same temporal signatures, suggesting that detailed quantification of the pattern of spikes in SpW discharges may be a useful indicator of disparate underlying epileptogenic pathologies.

**NEW & NOTEWORTHY** Spike-and-wave-type discharges (SpW) are a common feature in many epilepsies. Their electrographic manifestation is highly varied, as are available genetic clues to associated underlying pathology. Using computational and in vitro models, we demonstrate that distinct subtypes of SpW are generated by lamina-selective disinhibition or enhanced interlaminar excitation. These subtypes could be detected in at least some noninvasive patient recordings, suggesting more detailed analysis of SpW may be useful in determining clinical pathology.

## INTRODUCTION

Spike-and-wave discharges (SpW) are seen in a broad range of different epilepsies. They are a common feature in idiopathic generalized epilepsies (Panayiotopoulos, 2005) and are particularly associated with absences, where they dominate the EEG interictally and during seizures (Crunelli and Leresche 2002; Tenney and Glauser 2013). However, they also manifest in juvenile and adult myoclonic and generalized tonic-clonic seizures (Panayiotopoulos 2005). In this context they appear as large-amplitude waves, often with one or more spikes nested within this wave, so-called spike-and-wave discharges (SpW) (Crunelli and Leresche 2002). It is thought they form part of a continuum of pathologies ranging from benign partial epilepsies through to continuous SpW associated with sleep (Medeiros et al. 2010). SpW are also seen in focal epilepsies, particularly (though not exclusively) in extratemporal areas such as frontal, parietal, and occipital cortices (Takahashi et al. 2015; Taylor et al. 2003; Westmoreland 1998).

SpW are heterogeneous, and this attribute has been used to delineate seizure types (Blumenfeld 2005). Characterization of electrographic events in terms of the frequency of the wave component of SpW separates typical and atypical absences: SpW are manifest in EEG occurring at frequencies of 3–4 Hz (Gibbs et al. 1935) and 1–2 Hz (Niedermeyer 1968), respectively. Although some authors challenge delineation between these subtypes (Holmes et al. 1987), typical and atypical absences present with different levels of consciousness impairment, preictal EEG patterns, and spatial manifestations, particularly with respect to relative roles for neocortical and thalamic activity (Blumenfeld 2005). Detailed analysis of the waveform characteristics of single SpW events in other epilepsy subtypes also reveals considerable variability (Keller et al. 2010). The wave component of a SpW appears to correspond to a single period of delta or slow-wave oscillation seen in normal subjects during sleep (Kostopoulos 2000). This suggestion certainly correlates with the proposed generatory role for thalamus (Castro-Alamancos and Calcagnotto 1999). However, SpW were originally noted to be of cortical origin (Gibbs et al. 1935), and further work appears to support this (Seneviratne et al. 2014). In addition to the wave component, the manifestation of spikes associated with each wave event is also heterogeneous. The classic “dart and dome” profile (Lennox and Davis 1950) is complicated by different numbers of spikes per wave (Crunelli and Leresche 2002) and the temporal relationship between each spike and the underlying wave (Weir 1965).

Acute, in vitro animal models of epileptiform events have shed much light on the underlying pathophysiology of interictal discharges and tonic-clonic electrographic events (e.g., Hegstad et al. 1989), but little progress has been made using this approach to understand the origins of the above temporal and spatial differences. Support for a thalamic origin (e.g., see Niedermeyer 1996) comes from thalamic disinhibition in vitro. Blockade of GABA_A_ receptors has been shown to generate typical absence-like 3- to 5-Hz waves (without spikes) in relay neurons projecting to cortex (von Krosigk et al. 1993). In contrast, most experimental models show a lack of necessity for thalamic input for generating both delta and SpW (e.g., Carracedo et al. 2013; Hall et al. 2015). Bilateral cortical lesions abolish SpW in a genetic rat model (Scicchitano et al. 2015), leading these authors to suggest a purely neocortical origin for this type of epileptiform activity. In support of this, careful examination of in vivo recordings indicates a layer 5/6 origin with subsequent projection of seizure-like activity down to thalamus (Timofeev and Steriade 2004). More recent work has neatly demonstrated a specific role for layer 6 calcium channel expression in this propagation (Bomben et al. 2016). In terms of the spike component of SpW, there does appear to be consensus that these events originate in superficial layers of neocortex (Hall et al. 2015; Schwartzkroin et al. 1983; Ulbert et al. 2004).

Clues to the mechanism underlying SpW also come from genetic studies in patients, but, again, the evidence shows remarkable heterogeneity (e.g., see Noebels 2017). Correlations with mutations in GABRG2 and GRIN2A are reported (Crunelli and Leresche 2002). The former codes for the γ2-subunit of GABA_A_ receptors and is critical for the localization and maintenance of this vital component of synaptic inhibition (Schweizer et al. 2003). GRIN2A codes for the NR2A subunit of NMDA receptors, and gain-of-function mutations lead to epilepsy with marked language difficulties (Carvill et al. 2013). It is also thought to be highly predictive of syndromes which manifest with SpW, such as Landau-Kleffner and continuous spike-and-wave during sleep (Carvill et al. 2013). These data suggest that the relationship between underlying pathology and the type of SpW manifest in patients may be complex.

In the present study we focus on the two above-mentioned neuronal control systems (synaptic inhibition and NMDA receptor-mediated excitation) to further understand differential mechanisms underlying the heterogeneity of spike manifestation within SpW. Using experimental animal and computational models of physiologically relevant slow oscillations (nested delta and theta rhythms), we found that distinct patterns of multiple spikes per SpW seen in patients could be selectively generated by either predominantly superficial cortical layer disinhibition or enhanced ascending excitation.

## MATERIALS AND METHODS

### 

#### Rodent model.

All procedures were performed under license from the United Kingdom government and conformed to the regulations detailed in the UK Animals (Scientific Procedures) Act, 1986. Parietal coronal slices (450 μm thick) were prepared from adult male Wistar rats (~200 g) and maintained at 34°C in oxygenated artificial cerebrospinal fluid (ACSF) consisting of (in mM) 126 NaCl, 3 KCl, 1.25 NaH_2_PO_4_, 0.6 MgSO_4_, 1.2 CaCl_2_, 24 NaHCO_3_, and 10 glucose. Delta oscillations were generated as described previously (Carracedo et al. 2013). Bath application of (−)tubocurarine chloride (dTC; 10 μM) and/or BMS-193885 [10 μM; neuropeptide Y (NPY) receptor antagonist] and vasoactive intestinal peptide (VIP; 1 μM) were used to generate spike-and-wave-like epileptiform events (Hall et al. 2015). Drugs were obtained from Santa Cruz Biotechnology, Tocris Bioscience, or Sigma Aldrich.

All recordings were conducted in the secondary somatosensory cortex. Extracellular recordings were obtained using micropipettes (2–5 MΩ) filled with ACSF and were bandpass filtered at 0.1 to 300 Hz. Intracellular recordings used micropipettes (50–150 MΩ) filled with 2 M potassium acetate and were recorded DC to 2.5 kHz. Enhancement of layer 5 (L5) to layers 2/3 (L2/3) excitatory connections was performed by theta burst stimulation (TBS): 5 pulses at 50 Hz, repeated at 5 Hz for 10 s delivered to L5. The frequency of repetitive burst discharges in superficial cortical layers was analyzed from slices bisected at the level of layer 4. The resulting superficial layer-only slices were bathed in ACSF containing dTC and gabazine (1 μM).

#### Data analysis.

Local field potential, EEG, and magnetoencephalographic (MEG) spike detection was performed as follows: raw data traces were high-pass filtered at 1 Hz to remove the wave component of the spike-and-wave discharges. Time points corresponding to the peak negativity of each spike were recorded if the magnitude was >1 SD from the mean potential of each data epoch. Inhibitory (IPSP) and excitatory synaptic potential (EPSP) amplitudes were measured from −30 and −70 mV, respectively. Granger causality estimates were calculated from 30 raw data epoch pairs (field potentials from L2 and L5; 600-ms duration centered around peak L2 negativity) in each condition (delta, single spike-and-wave, or multiple-spikes-per-wave events) using the BSMART toolbox. MEG sensor data (*patient 2*) was extracted using SPM (www.fil.ion.ucl.ac.uk/spm/). All statistical comparisons were performed using unpaired, 2-tailed *t*-tests when single observations were taken for each slice used. When multiple observations per slice were included, we used analysis of variance with replicates. When more than two data sets were compared, we used analysis of variance with Bonferroni correction. If the data failed the equal variance test or were nonnormally distributed, we used the Mann-Whitney test. All tests were performed using SigmaStat (Systat, London, UK).

#### Computational model.

Spike-and-wave discharges were simulated on a background delta rhythm model (Traub et al. 2005) according to the parameters detailed in Hall et al. (2015). A review of the structure of this thalamocortical column model and the initial results are as follows: model neurons had a schematic structure, with dozens of compartments, including soma, a short axon, and branching dendrites. There were multiple active membrane conductances, including transient and persistent *g*_Na_, high- and low-threshold *g*_Ca_, a collection of K^+^ conductances [delayed rectifier, A and M types, and 2 Ca^2+^-dependent types corresponding to SK (small conductance) and BK (large conductance) channels], and an anomalous rectifier. The neuronal types and their numbers were as follows: superficial RS (regular spiking) pyramids, 1,000; superficial interneurons, 360 [partitioned into basket, axo-axonal, low-threshold spiking (LTS), and neurogliaform]; spiny stellates, 240; deep IB (intrinsic bursting), 2,000; deep RS, 500; deep interneurons, 400 (partitioned as above). Thalamic cells (relay, nucleus reticularis) were shut off. Spiny stellate cells, in our simulations, made minimal or no contribution to delta oscillations or related epileptiform activities.

Model neurons were interconnected by chemical synapses and by gap junctions. Principal cells activated AMPA and NMDA receptor-mediated currents in postsynaptic targets. All interneurons produced GABA_A_ currents (which had slower kinetics for LTS cells than for other interneurons); neurogliaform cells produced GABA_B_ currents, as well. Gap junctions occurred between axons of homologous principal cells and between dendrites of homologous types of interneurons. Heterogeneity and noise were introduced by spreads of bias currents to cell somata and by random, Poisson-distributed ectopic spikes occurring in the distal axons of principal cells.

In previous studies, delta oscillations were generated primarily by deep IB cells, recurrently interconnected with strong NMDA currents. Delta period length was primarily determined by GABA_B_ currents produced by neurogliaform cells. The firing pattern of deep RS pyramidal cells was influenced by the IB cells and also strongly depended on tonic membrane potential (Carracedo et al. 2013). SpW was produced (Hall et al. 2015) from delta by relative disinhibition among superficial RS cells, with details of the pattern influenced by RS cell membrane potential, recurrent excitation in superficial layers, and synaptic excitation from deep layers (either RS or IB cells, in different simulations). These parameters were further explored in the present study (see below).

The database for the combined study of delta, SpW, and multiple spikes per wave consisted of over 500 simulations, with over 100 used for the present report. Because of the complexity of the model and the length of the simulations (see below), a complete exploration of the parameter space could not be contemplated, even after an attempt to fix as many parameters as possible from published literature (see Traub et al. 2005). The model should be viewed not as reality, but instead as a tool for testing physiological intuitions.

For the present study, long- and short-latency multiple-spikes-per-SpW events were specifically modeled by, respectively, *1*) increasing excitatory synaptic conductance from deep RS to superficial RS cells (via a 2.5-fold increase in connectivity and a 5-fold increase in unitary AMPA receptor conductance) and *2*) additionally reducing GABA conductances in superficial RS cells. Control simulations (not shown) demonstrated that increasing excitatory conductance was not necessary to obtain short-latency multiple spikes, as long as there was superficial disinhibition. Superficial disinhibition was accomplished by reducing GABA_A_ inputs to RS cells 3-fold from superficial basket cells and 8-fold from superficial neurogliaform cells and by reducing GABA_B_ inputs to RS cells 14-fold. As before, programs were written in Fortran to run on 24 nodes of an IBM parallel server, AIX operating system (IBM’s version of Linux), with the message passing interface environment used to handle communication between nodes. Simulations of 5 s of “neuronal time” lasted up to 22 h. All Fortran code is available in ModelDB (http://modeldb.yale.edu/235561).

#### Human and human tissue studies.

Illustrative data from three patients with different diagnoses are presented to suggest possible clinical relevance for the in vitro observations. For *patient 1*, EEG data were from a child (age 10 yr) presenting from age 6 yr with early morning seizures. Continuous SpW discharges were observed at this time, leading to a diagnosis of electrical status epilepticus in sleep. For *patient 2*, MEG data were from a child (age 7 yr) with idiopathic generalized epilepsy who had nocturnal seizures of increasing frequency since age 4 yr. Seizures manifested as a mixture of spike-and-wave discharges, polyspikes, and, again, protracted continuous SpW discharges. For *patient 3*, left temporal neocortical tissue was obtained from a 55-yr-old male patient. The patient had suffered from seizures of a predominantly temporal lobe origin for a 30-yr period. Tissue was recorded in the same in vitro conditions as those in rodents (above).

## RESULTS

### 

#### Computational model predicted roles for superficial disinhibition and interlaminar excitation in shaping spike-and-wave discharges.

We first used a computational model of cognitively relevant delta/theta rhythms (Carracedo et al. 2013) and enhanced L5-to-L2/3 excitatory connectivity (Feldmeyer 2012). Ascending excitatory projections from L5 to supragranular layers have been shown to be potent (Holmgren et al. 2003) with a late component exquisitely sensitive to degree of NMDA receptor-mediated synaptic excitation on repeated activation (Cauller and Connors 1994) and thus likely to be enhanced in the gain-of-function mutations seen in some patients and animal models (Carvill et al. 2013; Lacey et al. 2012).

**Fig. 1.**
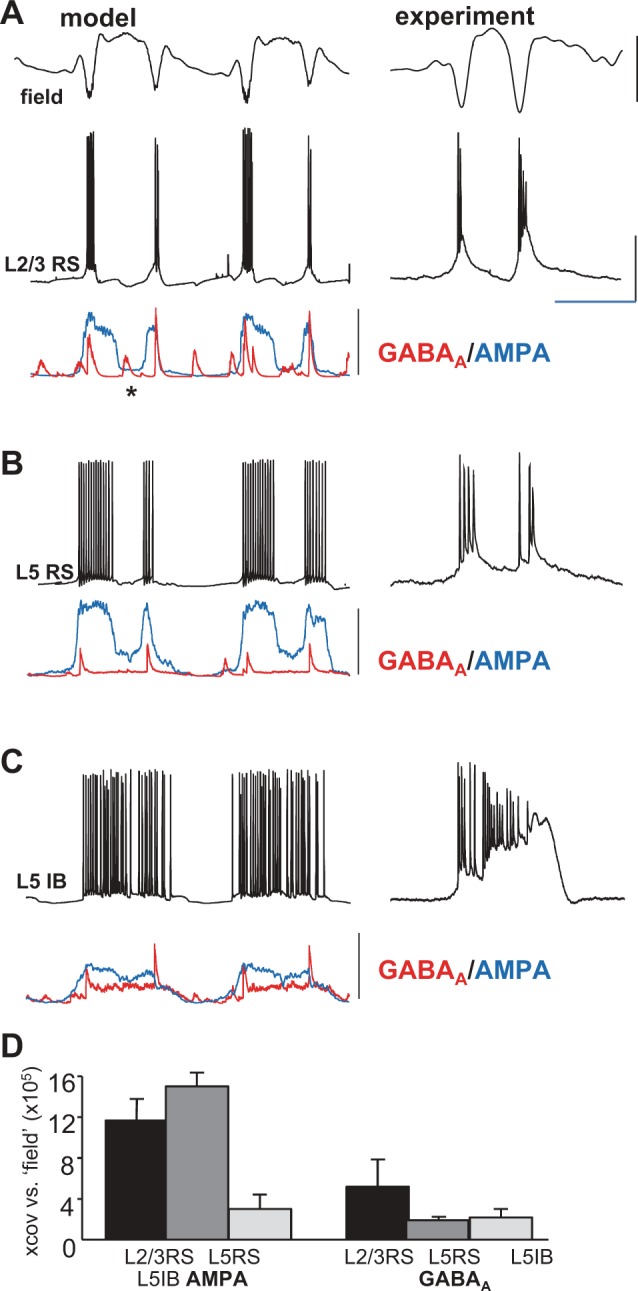
The computational model predicted enhanced L5–L2/3 synaptic excitation was alone sufficient to generate long-interval multiple spikes per wave. *A*, *left*: example time series from SpW simulations (see Hall et al. 2015 for details) in which only the conductance of excitatory synaptic connections from L5 RS (regular spiking) neurons to L2/3 RS neurons was increased. Simulated superficial cortical field potential (model) was derived from the sum of all synaptic and intrinsic voltage changes in L2/3 RS cells and the inverted sum of all voltage changes in L5 neurons. A somatic membrane potential time series from a single neuron (L2/3 RS) is illustrated with concurrently simulated GABA_A_ (red) and AMPA (blue) input conductances. Note the long-latency double bursts corresponding to the field double spikes per wave event (2 events shown). Each burst was accompanied by mixed synaptic input (inhibition and excitation), whereas additional, shorter latency activity (not expressed in the field) in L2/3 was suggested by the isolated inhibitory events in this neuron subtype (asterisk). *Right*, one multiple-spikes-per-wave event from intracellular recordings in the superficial layer in the corresponding experimental model (TBS). Scale bars: arbitrary (model field), 0.5 mV (experimental field), 25 mV (L2/3 RS), 15 nS (GABA_A_), 30 nS (AMPA); 150 ms. *B* and *C*, *left*: concurrently simulated membrane potential and synaptic input conductances from an L5 RS (*B*) and L5 IB neuron (*C*). *Right*, corresponding intracellular membrane potential data for individual multiple spikes-per-wave events in the TBS experimental model (see Fig. 5). Note experimental traces in *A–C* were not concurrently recorded. Scale bars as in *A*. *D*: mean (±SE) nonnormalized cross-covariance maxima for each model SpW are illustrated to quantify the relative contribution of each cell’s excitatory and inhibitory synaptic inputs to the shape of the observed model field. Compared with the model field, excitatory and inhibitory conductance profiles were tested for each of the 3 principal cell subtypes modeled.

On a baseline of parameters producing single spikes per wave from delta rhythms (see Hall et al. 2015), increasing excitation of L2/3 regular spiking (RS) by L5 RS cells (see *Computational models*) generated pairs of field spikes per SpW (Fig. 1*A*). The interspike interval was 146 ± 9 ms (*n* = 5 model SpW simulations). The pairs of spikes in the simulated field were associated with brief, intense bursts in L2/3 RS cells, in turn associated with large, compound excitatory postsynaptic conductance increase. Cross-covariance analysis of this measure of synaptic excitation showed high temporal correlation with the model field (Fig. 1, *A* and *D*; 11.5 ± 2.5, *n* = 5 SpW events). Similarly, cross-covariance of the synaptic excitation profile during SpW in L5 RS cells with the field also yielded high values (Fig. 1, *B* and *D*; 15.1 ± 1.1, *n* = 5). L5 RS cell bursts of action potentials began 6 ± 2 ms before L2/3 RS bursts (*n* = 10 events). In contrast, cross-covariance values for the model field and the prolonged synaptic excitation profile in L5 intrinsically bursting (IB) neurons were relatively poor (Fig. 1, *C* and *D*; 3.0 ± 0.8, *P* < 0.05). To determine the relative contributions of the excitatory and inhibitory synaptic inputs to each cell type to the observed shape of the field potential, cross-covariance of model inhibitory synaptic conductance changes in each of the principal cells modeled compared with the model field also yielded low values (Fig. 1*D*).

**Fig. 2.**
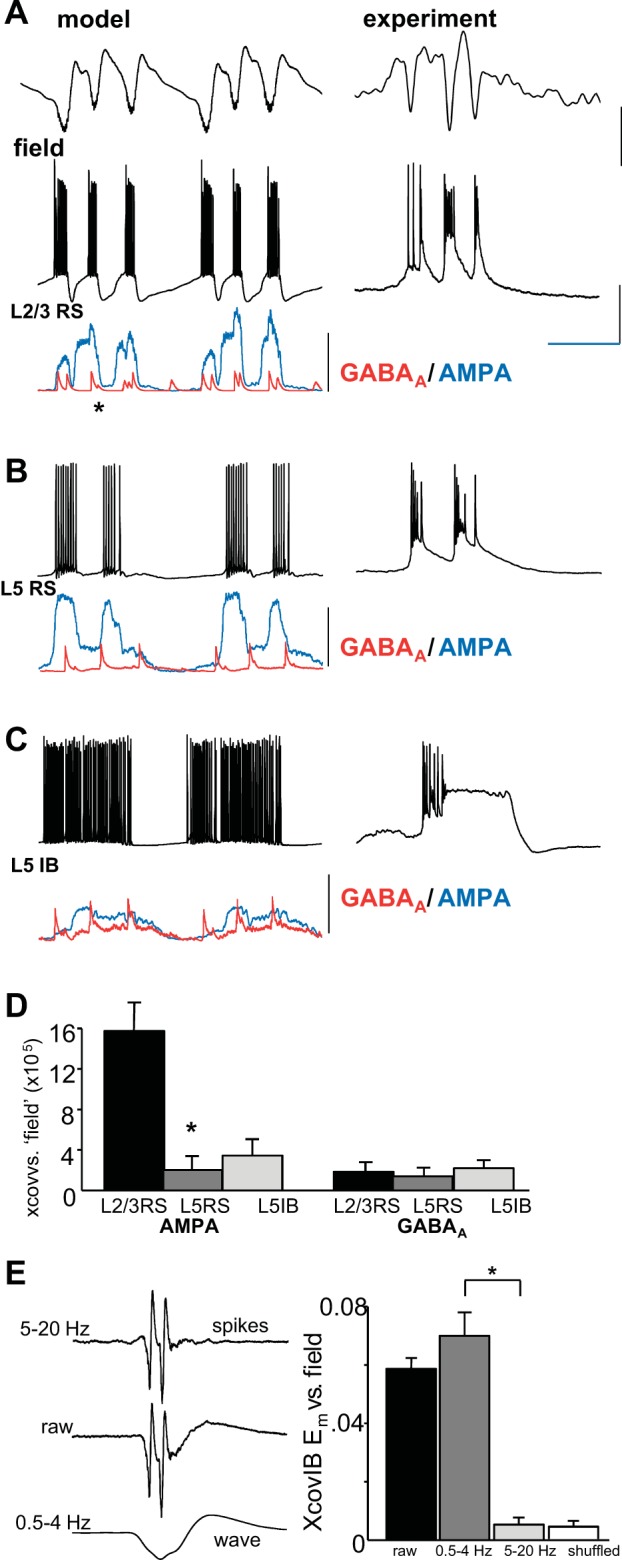
The computational model predicted L2/3 disinhibition was alone sufficient to generate short-interval multiple spikes per wave. *A*, *left*: example time series from SpW simulations (see Hall et al. 2015 for details) in which the conductance of inhibitory synaptic connections onto L2/3 RS neurons was decreased. Simulated superficial cortical field potential (model) was derived as in Fig. 3. A somatic membrane potential timeseries from a single neuron (L2/3 RS) is illustrated with concurrently simulated GABA_A_ (red) and AMPA (blue) input conductances. Note the short-latency triple bursts corresponding to the field triple-spikes-per-wave event (2 events shown). Each burst was accompanied by mixed synaptic input (inhibition and excitation), and much reduced, isolated inhibitory events in this neuron subtype were seen during the spike-and-wave events (cf. Fig. 3*A*). *Right*, intracellular recordings of a single multiple-spikes-per-wave event from superficial layer in the corresponding experimental model (peptidergic disinhibition). Scale bars: arbitrary (model field), 0.5 mV (experimental field), 25 mV (L2/3 RS), 15 nS (GABA_A_), 30 nS (AMPA); 150 ms. *B* and *C*, *left*: concurrently simulated membrane potential and synaptic input conductances from an L5 RS (*B*) and L5 IB neuron (*C*). *Right*, corresponding intracellular membrane potential data for individual spike-and-wave events in the peptidergic disinhibition model. Note experimental traces in *A–C* were not concurrently recorded. Scale bars as in *A*. *D*: mean (±SE) nonnormalized cross-covariance maxima for each model SpW. Compared with the model field, excitatory and inhibitory conductance profiles were tested for each of the 3 principal cell subtypes modeled. Asterisk denotes significant difference compared with corresponding simulation values for the enhanced excitation model (Fig. 1). *E*: mean (±SE) nonnormalized cross-covariance maxima for L5 IB membrane potential and concurrent L5 field potential. Covariances were calculated from 20 events each from *n* = 4 neurons for the unfiltered field (raw), the high-pass-filtered field showing spikes only, and the low-pass-filtered field showing waves only. *P < 0.01.

We then modeled the disinhibition suggested by GABRG2 mutations in some patients predominantly in superficial cortical layers as suggested by pial disinhibition (Schwartzkroin et al. 1983) and selective neuropeptide-mediated disinhibition (Hall et al. 2015). Decreasing the conductance driving model GABA_A_ receptor-mediated IPSPs only onto superficial RS neurons transformed SpW with single field spikes into SpW with triplet spikes. These multiple spikes per wave had significantly shorter interspike intervals than those seen with the enhanced excitation model described above (cf. Fig. 1*A*, Fig. 2*A*; *P* < 0.05). Interspike interval was 81 ± 4 ms (*n* = 5 model SpW events). In this variant of the model, each spike in the field was again associated with a brief burst in L2/3 RS neurons, in turn associated with a large, compound excitatory conductance change (Fig. 2*A*). Cross-covariance analysis of the temporal profile of this measure of synaptic excitation with the model field showed high correlations (15.6 ± 3.1, *n* = 5) not significantly different from those observed for this measure in the enhanced excitation model (cf. Fig. 1*D*, Fig. 2*D*; *n* = 5, *P* > 0.1, repeated-measures ANOVA of peak covariance values with Bonferroni correction for repeat comparisons). This strongly suggested that L2/3 RS neuron excitation was the source of the spikes observed in the model field potential. In contrast, comparing cross-covariance values for the model field with the deeper L5 RS cell’s synaptic excitation during superficial disinhibition revealed significantly less correlation compared with the enhanced excitation model (cf. Fig. 1, *B* and *D*, Fig. 2, *B* and *D*; *P* < 0.05). This suggested a “disconnect” between superficial and deep layer RS neurons during superficial disinhibition that was not seen with enhanced deep–superficial excitation. Overall timing of all L5 bursts gave a highly variable L5-to-L2/3 onset time of 12 ± 32 ms (*n* = 10). Excitatory input to L5 IB cells and inhibitory inputs to each of the three principal cells modeled also showed low cross-covariance values compared with the model field (Fig. 2*D*).

**Fig. 3.**
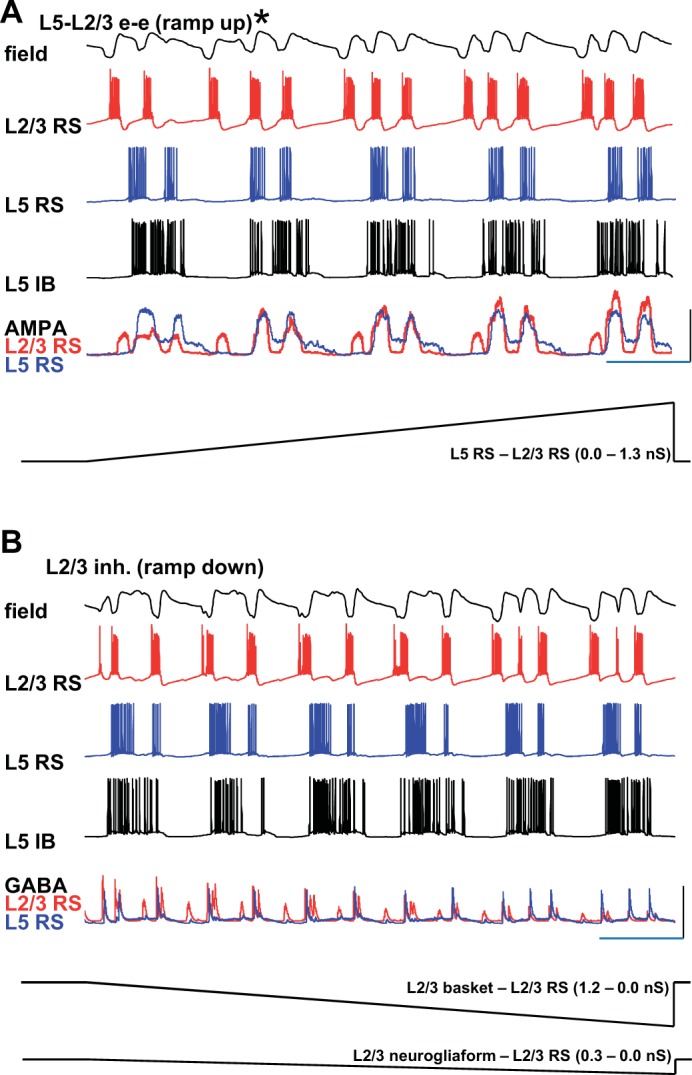
Combined L5*–*L2/3 excitation and L2/3 disinhibition generate mixed long- and short-interval spikes in a parameter-dependent manner. *A*: example computer simulation of SpW during continuously increasing values of L5*–*L2/3 excitation from a baseline of L2/3 disinhibition as used in Fig. 1. Asterisk denotes a single spike-and-wave event where both short- and long-latency spikes are seen riding on the same wave. Note L5 RS and IB neuronal outputs remain unchanged throughout, but L2/3 RS spiking and the simulated field progress from long-interval spikes in each SpW event to short-interval spikes only. Scale bars: arbitrary (field), 100 mV (L2/3 RS, L5 RS, L5 IB), 30 nS (AMPA); 500 ms. *B*: example computer simulation of SpW during continuously decreasing values of L2/3 inhibition from a baseline of L5*–*L2/3 excitation used in Fig. 3. Note, again, that L5 RS and IB neuronal outputs remain unchanged throughout and that L2/3 RS spiking and the simulated field progress from long-interval spikes in each SpW event to short-interval spikes only. Scale bars as in A, except 15 nS (GABA_A)_.

The two computational models thus far simulated either long- or short-interval multiple spikes per SpW only. To understand whether combinations of parameters could generate both multiple spike signatures, we combined both the elevated L5-to-L2/3 excitation conditions (Fig. 1) with the selective L2/3 disinhibition (Fig. 2) model parameters. Starting with partial disinhibition, unitary excitatory conductance for L5 RS-to-L2/3 RS model neurons was monotonically increased from 0 to 1.3 nS. This transformed long-interval multiple spikes per SpW into short-interval multiple spikes (Fig. 3*A*). Over a very narrow range of interlaminar excitation values, coexistence of long- and short-interval spikes was seen on a single SpW event (Fig. 3*A*, asterisk). Conversely, starting with elevated synaptic excitation from L5 RS cells to L2/3 RS cells, a monotonic decrease in synaptic inhibition from superficial basket cells and superficial neurogliaform cells to superficial RS cells (1.2 nS to 0 and 0.3 nS to 0, respectively) caused an abrupt change from long- to short-interval multiple spikes (Fig. 3*B*). Thus the nature of spikes observed on each SpW event appeared exquisitely sensitive to the balance of deep–superficial layer excitation and superficial layer disinhibition.

#### Experimental reproduction of heterogeneous spike-and-wave events.

**Fig. 4.**
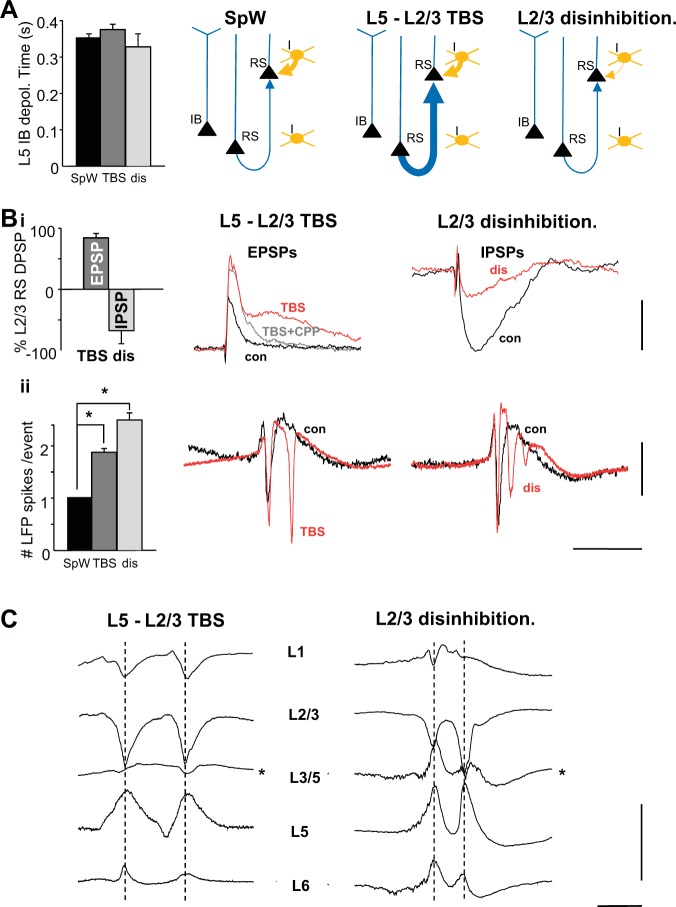
Experimental models of long- and short-interval multiple spikes per wave. *A*, *left*: the 3 model conditions used did not have any significant effect on the duration of L5 IB cell depolarization [depol.; SpW, bath application of (−)tubocurarine chloride (dTC); TBS, theta burst, tetanic stimulation of L5; dis, L2/3 disinhibition (bath application of BMS-193885 and VIP)]. *Right*, illustrations of the basic network weight changes in each model compared with control (I, interneuron). *Bi*: TBS generated almost double the amplitude of electrically stimulated L5–L2/3 EPSPs in RS cells (*left*; *P* < 0.05, *n* = 20 replicates from *N* = 5 slices) and exposed an overt NMDA receptor-mediated later component (*middle*). Peptidergic disinhibition caused a near halving of monosynaptic IPSP amplitude onto L2/3 RS cells (*P* < 0.05, *n* = 10 replicates from *N* = 5 slices). *Middle*, average (*n* = 10) example intracellular traces (resting membrane potential = −70 mV) showing the change in L5–L2/3 EPSP amplitude and profile. *Right*, shows average (*n* = 10) intracellular example traces (resting membrane potential = −30 mV) in the presence of (*R*)-CPP (10 μM) and NBQX (20 μM) to illustrate the reduction in electrically stimulated superficial layer IPSPs onto L2/3 RS cells. *Bii*: graph showing mean (*n* = 50) number of spikes per wave in each of the 3 conditions illustrated. Note dTC alone (SpW) generated only single spikes per wave in all incidences. TBS generated between 1 and 2 spikes per wave, and superficial layer disinhibition (dis) generated between 2 and 3 spikes per wave. *P < 0.05; *n* = 10 SpW events from *N* = 5 slices. Example L2/3 LFP traces (*middle* and *right*) show the difference in interspike interval for the two multiple-spikes-per-wave models, compared with control (con). Note TBS generated only long-interval multiple spikes, whereas disinhibition generated only short-interval multiple spikes per wave (see Fig. 5). *C*: laminar profile of SpW events in the two models. *Right*, example SpW events with dual spikes in 1 slice from the TBS condition. Both wave and spike components reversed when deep and superficial laminae were compared. *Left*, SpW events with dual spikes in 1 slice from the disinhibition model. Again, both spike and wave components reversed. Note in middle layers that the magnitude and direction of spike components was variable (asterisk). Scale bars: 6 mV, 60 ms (*Bi*); 0.4 mV, 300 ms (*Bii*); 1 mV, 100 ms (*C*).

The computational model predicted the mechanism(s) underlying long- and short-interval multiple spikes per SpW involved ascending interlaminar excitation increases and selective superficial layer disinhibition, respectively. To experimentally test these predictions, we first used theta burst stimulation (TBS) of L5 to potentiate ascending excitatory connections and to mimic the gain of function in NMDA-dependent neurotransmission associated with SpW generation (Carvill et al. 2013). Second, we shifted the balance of predominantly superficial neocortical layer peptidergic inhibition away from NPY receptor dominance and toward VIP receptor dominance (Fig. 4*A*; bath application of BMS-193885 and VIP) to induce a predominantly superficial layer state of partial disinhibition (Hall et al. 2015).

TBS generated an 85 ± 7% increase in L2/3 pyramidal cell excitatory postsynaptic potentials (EPSPs) evoked by single electrical stimulation (Fig. 4*Bi*; *P* < 0.05, pre- vs. post-TBS, *n* = 20 replicates from *N* = 5 slices, paired *t*-test). In addition, the potentiated EPSPs also possessed an additional slow component reaching a maximum 37 ± 5 ms after the initial peak that was not seen in pre-TBS conditions. This slow component was selectively blocked by bath application of the noncompetitive NMDA receptor antagonist (*R*)-CPP (10 μM) [TBS vs. TBS + (*R*)-CPP, *P* > 0.1, paired *t*-test (initial EPSP magnitude), *P* < 0.05 (late EPSP magnitude); Fig. 4*Bi*]. These changes were not accompanied by changes in the L5 IB cell activity that underlies the “wave” component of the field spike-and-wave discharge (Hall et al. 2015). No significant change in depolarization peak magnitude (data not shown) or duration (Fig. 4*A*) was seen when the SpW model alone was compared with the SpW model post-TBS (*P* > 0.1, *n* = 10 events from *N* = 5 slices, *t*-test), suggesting this experimental manipulation did not affect L5-L5 IB cell synaptic excitatory events. Despite this, the number of field spikes in each SpW increased from the stereotypical single spike per wave to an average of 1.8 ± 0.2 spikes per wave post-TBS (*P* < 0.05, pre- vs. post-TBS, *n* = 10 SpW events from *N* = 5 slices, paired *t*-test; Fig. 4*Bii*).

The disinhibition model produced a 69 ± 24% decrease in monosynaptic inhibitory postsynaptic potentials (IPSPs) in L2/3 pyramidal cells (*P* < 0.05, pre- vs. post-BMS-193885 + VIP, *n* = 10 events from *N* = 5 slices, paired *t*-test; Fig. 4*Bi*) with no significant change in IPSP decay constant (22 ± 4 vs. 20 ± 6 ms, pre- vs. post-BMS-193885 + VIP, *P* > 0.1, *n* = 10 events from *N* = 5 slices). This decrease in superficial layer synaptic inhibition was also not accompanied by significant changes in the intensity of the L5 IB cell discharges (*P* > 0.05, *n* = 10 events from *N* = 5 slices; Fig. 4*A*). However, a 1.5-fold increase in the number of spikes observed on average per SpW event was seen (*P* < 0.05, *n* = 10 events from *N* = 5 slices; Fig. 4*Bii*).

The observation of multiple spikes did not depend on which layer data were recorded from (Fig. 4*C*). In each case, a clear reversal of both spike and wave components was seen when superficial and deep recordings were compared. However, recordings from mid-cortical layers poorly resolved the typical shape of the SpW and, in addition, led to differential polarity of spikes, even within a single SpW event (Fig. 4*C*, asterisk).

#### Temporal profile of multiple spikes correlated with superficial and deep layer network time constants.

**Fig. 5.**
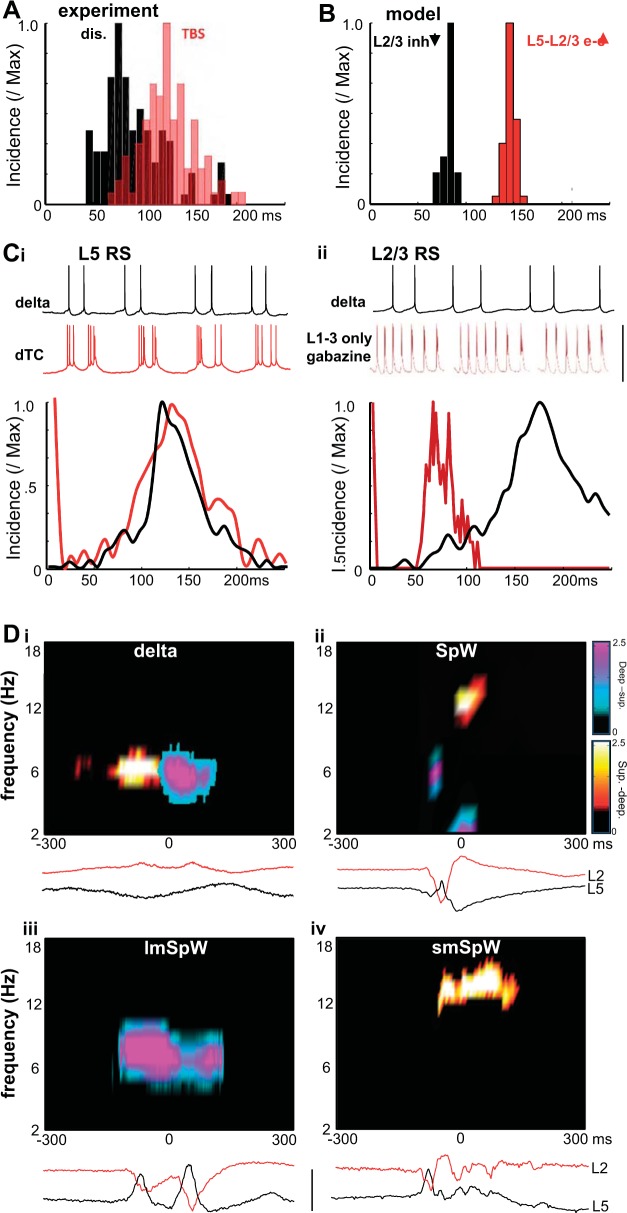
Long- and short-interval multiple spikes correlate with time constants in L5 and L2/3 RS cell circuits, respectively, and differentially disrupt interlaminar communication. *A*: histogram showing the incidence of interspike intervals (from 100 temporally adjacent peak negativities) for multiple-spikes-per-wave activity occurring in the experimental TBS model (red) and the superficial disinhibition model (dis; black). *B*: histogram showing the incidence of interspike intervals for computational model activity occurring with elevated L5–L2/3 excitation (e-e; red) and L2/3 disinhibition (inh; black). *Ci*: 1.5-s- duration intracellular recordings from L5 RS neurons during normal delta rhythm (black) or in the presence of dTC (red). Graph shows incidence of L5 RS intracellular interspike intervals for 50 delta (black) or SpW (dTC) events (red). Note no field spikes or only single field spike-per-wave events were generated in the delta or dTC-alone conditions, respectively. Despite this, interspike/interburst interval histograms show a peak corresponding to long-latency multiple-spikes-per-wave events. *Cii*: black trace shows 1.5-s-duration example of spiking in L2/3 RS neurons during normal delta rhythms. Dark red trace shows L2/3 RS rhythmic burst discharges generated in superficial layer-only slices (deep layers removed at the level of L4) in the presence of dTC and disinhibition (1 μM gabazine). Three burst discharge trains from the same neuron are shown with the initial “tonic” component of the spontaneous epileptiform discharges removed for clarity. Graph shows incidence of L2/3 RS intracellular spikes during delta rhythm (black) and during superficial network-only, repetitive burst discharges (dark red). Note L2/3 spiking during delta rhythms did not correspond with either long- or short-latency spike intervals during multiple-spikes-per-wave discharges, but repetitive, superficial layer-only bursting, during disinhibition, matched the short-interval multiple-spikes-per-wave time constant. Scale bars: 80 mV, 300 ms. *Di*: Granger causality estimates of the influence of superficial layer (L2) activity on deep layers (L5) (hot colormap) and deep layer activity on superficial layers (cool colormap) during normal experimental model delta activity. Note the bidirectional interaction at theta frequencies (see Carracedo et al. 2013). *Dii*: bidirectional laminar interactions revealed by Granger estimates for SpW events. *Diii*: Granger scores for long-latency (TBS model) multiple-spikes-per-wave events. *Div*: Granger scores for short-latency (superficial disinhibition model) multiple-spikes-per-wave events. Note the absence of bidirectional laminar interactions in *Diii* and *Div*. Paired example traces show concurrently recorded field potentials from L2 and L5 in each of the conditions shown. Scale bar: 0.5 mV; timescale is the same as the colormap *x*-axes.

The two experimental and computational models suggested different interspike intervals within SpW events depending on the generator mechanism: superficial disinhibition or enhanced excitation. We explored this further by comparing interspike intervals in the two experimental models with interevent intervals in deep and superficial layers in different conditions. With TBS, the interspike interval distribution peaked at bin center 125 ms (mean interval 124 ± 8 ms; *n* = 88 events from *N* = 5 slices; Fig. 5), whereas a highly significant difference was observed in the disinhibition model (*P* < 0.001, *n* = 88 for TBS, *n* = 109 for peptidergic disinhibition, Mann-Whitney rank sum test) in the distribution peaked with shorter intervals with bin center 75 ms (mean interval 84 ± 7 ms). Together, the two distributions appear to show a very good fit to the two interspike interval peaks seen in the computational models of disinhibition and TBS (Fig. 5*B*). No significant differences were found for either the short- or long-interval spike separation when data from the computational model and the experimental model were compared (*P* > 0.1, *n* = 10 events).

The longer field interspike intervals seen in the experimental and computational model SpW matched interevent intervals in L5 RS neurons (Fig. 5*Ci*). During delta rhythms (the primary substrate for SpW), L5 RS neurons fire doublets at approximately theta frequency (Carracedo et al. 2013). This pattern of doublets was transformed into double burst discharges in the tubocurarine model of SpW with single field spikes. The distribution of overall interspike intervals (*n* = 50 delta or SpW events from *N* = 5 slices) in each type of L5 RS output (pairs of spikes or pairs of bursts) revealed a peak not significantly different from that seen for long-interval field spikes from experimental and computational TBS models (*P* > 0.1, repeated-measures ANOVA with Bonferroni correction). Thus the longer interval, multiple spike dynamics appeared to correspond to the natural theta frequency observed in L5 RS cells during physiological delta rhythms (Carracedo et al. 2013).

In contrast, no comparable time constant could be found for deep neuronal (RS or IB) spike or burst outputs and the shorter interval field spikes seen in the partial disinhibition model (data not shown). This suggested the shorter field interspike interval in this type of SpW may have reflected time constants in superficial cortical layers. To test this, we completely isolated superficial layers (a cut though layer 4). In these superficial mini slices, disinhibition generated repeated polyspike-like discharges with a mean interburst interval in L2/3 RS cells of 68 ± 7 ms (*n* = 28). This was not significantly different from the shorter interspike interval seen in the peptidergic disinhibition model in full neocortical slices during SpW (*P* > 0.1, *n* = 25 events from *N* = 4 slices, repeated-measures ANOVA; Fig. 5*Cii*).

#### Different spike-and-wave events had different effects on local interlaminar communication.

Physiologically relevant models of cortical delta/theta nested rhythms revealed a role in controlling interlaminar communication (Carracedo et al. 2013). The relative timing of action potentials in L5 and L2/3 RS neurons revealed an iterative pattern of information transfer from superficial to deep and back to superficial layers. The data described above suggested that derangement of this combination of rhythms to generate SpW containing single and multiple field spikes per wave may disrupt the normal pattern of interlaminar cortical dynamics.

To test this we, estimated causal interactions between deep and superficial layers. Mean Granger causality estimates were calculated from concurrent L2/3 and L5 field potentials for *n* = 30 events of 4 types: *1*) single periods of the physiological delta rhythm (delta), *2*) spike-and-wave events showing stereotypical single field spikes (SpW; see Hall et al. 2015), *3*) spike-and-wave events showing multiple spikes with long (theta frequency) interspike interval (lmSpW; the TBS model), and *4*) spike-and-wave events showing multiple spikes with short interspike intervals (smSpW; the peptidergic disinhibition model) (Fig. 5*D*). As with previous experiments, the delta rhythm generated a clear pattern of iterative superficial–deep–superficial causality estimates occurring at theta frequency (Fig. 5*Di*). Disruption caused by each of the three subtypes of SpW was most apparent in this frequency range for superficial layer activity causal to deep layer activity: mean maximal Granger scores within the 4- to 8-Hz range were 2.6 ± 0.6 (delta), 0.5 ± 0.2 (SpW, one spike), 0.5 ± 0.3 (SpW long-interval multiple spikes), and 0.2 ± 0.1 (SpW short-interval multiple spikes) (*P* < 0.05, *n* = 30, delta vs. each SpW subtype, *N* = 5 slices, repeated-measures ANOVA with correction for multiple comparisons).

For interactions where deep layer activity was causal for superficial layer activity, the pattern of disruption was somewhat different: mean maximal Granger scores within the theta frequency range were 2.8 ± 0.2 (delta), 1.6 ± 0.5 (SpW, one spike), 2.4 ± 0.6 (SpW long-interval multiple spikes), and 0.1 ± 0.1 (SpW short-interval multiple spikes). Only the SpW subtype with short-interval multiple spikes (Fig. 5*Div*) showed a significant reduction in interlaminar interactions (*P* < 0.05, repeated-measures ANOVA with correction for multiple comparisons, *n* = 30, *N* = 5, delta vs. each subtype). Further differences between the long- and short-interval SpW events could be seen when a wider frequency range was considered (Fig. 5, *Diii* vs. *Div*). A dominant superficial layer to deep layer causality score was seen at low-beta frequencies (12–18 Hz) in the latter case.

#### Human spike-and-wave events can also be electrographically heterogeneous.

**Fig. 6.**
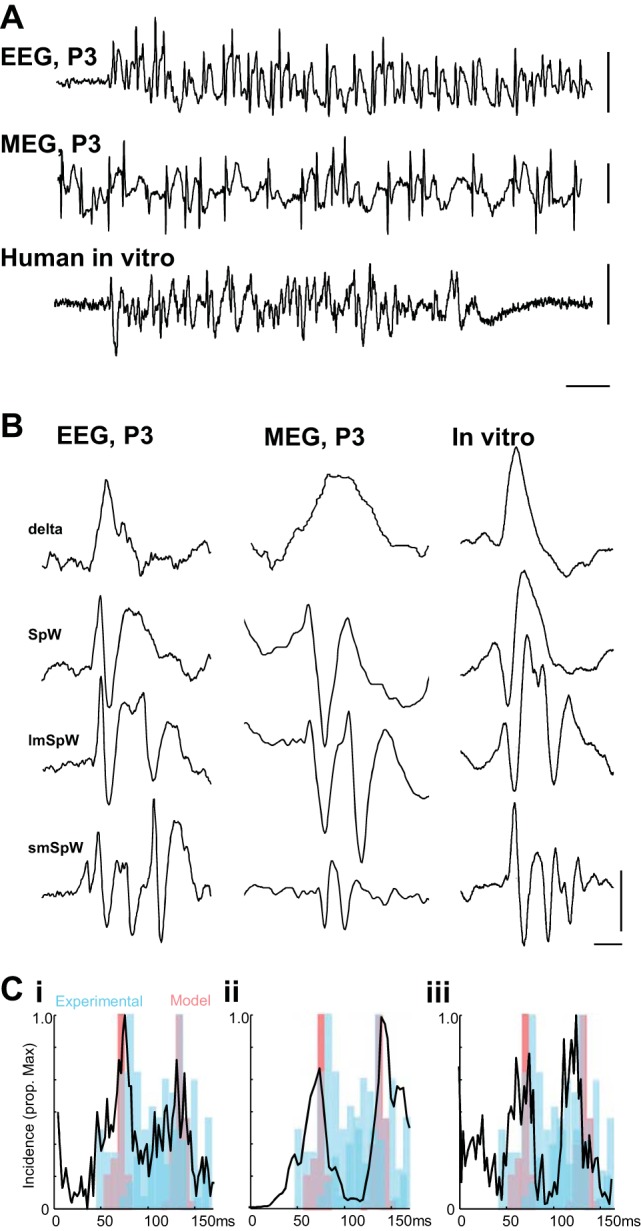
Four distinct subclasses of epileptiform event can be seen in patients with epilepsy. *A*: example noninvasive recordings from *patient 1* (EEG), *patient 2* (MEG), and spontaneous epileptiform discharges from L2/3 of a neocortical slice maintained in vitro from *patient 3* (human in vitro) showing the variety of electrographic events seen. P3 refers to the left parietal electrode in the 10-20 EEG system and equivalent sensor in the MEG recording apparatus. Scale bars: 0.5 mV (EEG), 4,000 fT (MEG), 1 mV (in vitro); 2 s. *B*: individual examples of 4 distinct epileptiform events in each of the 2 patients and patient tissue. In descending order, the events are described as large single wave (delta), spike-and-wave discharge (SpW), long-interval multiple spikes per wave discharge (lmSpW), short-interval multiple spikes per wave discharge (smSpW). Scale bars: 250 μV (EEG), 1,000 fT (MEG), 0.5 mV (in vitro); 100 ms. *C*: normalized incidence histograms of interspike intervals (from peak negativity) for >100 events in each example. Note the bimodal distribution of interspike intervals in each case. Data from experimental and computational models are superimposed in blue and red, respectively.

In each of the three human data sets (Fig. 6), two types of epileptiform discharge were seen. First, interictal events consisting of single SpW discharges. Second, ictal-like events lasting 1–7 min (*patient 1*, EEG; Fig. 6*A*), 1–20 s (*patient 2*, MEG; Fig. 6*A*) and 0.5–1 min (*patient 3*, temporal neocortical slice in vitro; Fig. 6*A*) consisted of heterogeneous SpW discharges. Four different types of discharge were seen (Fig. 6*B*): *1*) wave-only events, *2*) SpW with single spikes, *3*) SpW with pairs of spikes with interspike interval >100 ms, and *4*) SpW with 2–3 spikes with interspike interval <100 ms. These observed classes of SpW were not part of a continuously variable discharge pattern: interspike intervals showed a clear bimodal distribution with peaks at 74 ± 5 and 128 ± 7 ms for tissue from *patient 1*, 72 ± 7 and 142 ± 8 ms for tissue from *patient 2*, and 75 ± 11 and 125 ± 9 ms for tissue from *patient 3*. The pairs of interspike intervals observed were significantly different in each of the three recording modes/patients (*P* < 0.05, *n* = 20–50 events, *t*-test; Fig. 6*C*). No significant difference was seen between the long- and short-interval event peak frequencies in these three human examples and those seen in the experimental and computational models described above (*P* < 0.05, ANOVA with Bonferroni correction for multiple comparisons).

#### Different spike-and-wave events can have different origins and propagation profiles in human neocortex: an exemplar.

The above experimental and computational models suggested that both short and long interspike interval SpW subtypes observed in human recordings could occur in the same, small region of neocortex, the parietal cortex isolated in vitro. However, the presence of different types of SpW had dramatic effects on interlaminar communication in this region (Fig. 5*D*). Different cortical laminae project and receive activity from other cortical and subcortical regions differently. In addition, it has been shown that reduced inhibition has a dramatic effect on the speed of propagation of epileptiform activity across cortex. Reduced synaptic inhibition (as in the peptidergic disinhibition model used here) increases propensity of seizure-like activity to spread and dramatically affects the speed of that spread (Hall and Kuhlmann 2013; Trevelyan et al. 2007). From this, the models used in the present study suggested that SpW with short-interval multiple spikes should propagate faster than their long-interval SpW variants. We therefore used the noninvasive human data sets to investigate more global patterns of occurrence of disinhibition-based and excess excitation-based SpW subtypes predicted from the above-described interspike interval differences.

**Fig. 7.**
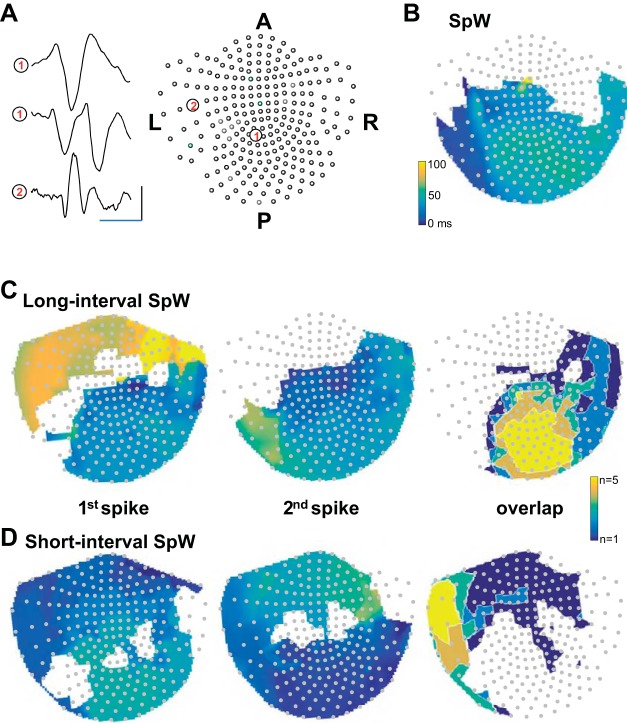
Long- and short-latency multiple spikes may originate and propagate in neocortex differently: a single patient example. *A*: example sensor data showing the 3 identified subtypes of SpW and the sensor positions from which they were taken, overlaid on the SPM analysis program sensor map for *patient 2*. Scale bar: 1,000 fT, 100 ms. *B*. map of significant negative peaks associated with single-spike-per-wave events. Colormap indicates time (in ms) from occurrence of 1st spike peak per event. Note the spikes occur first on left frontotemporal sensors and rapidly spread posteriorly to parieto-occipital sensors. *C*, *left*: map of significant negative peaks associated with long-interval multiple spikes per wave. Colormap as in *B*. *Right*, sensor positions demonstrating both spikes per wave (yellow area). *D*, *left*: map of significant negative peaks associated with short-interval multiple spikes per wave. *Right*, sensors demonstrating the first 2 spikes per event. Note the slower propagation of long-interval spikes compared with short-interval spikes and the almost complete lack of overlap between sensors showing either of these 2 SpW subtypes.

MEG sensor data showed SpW with single spikes were seen to originate almost simultaneously in frontal and left lateral regions before rapidly propagating (20–50 ms) to central parietal and occipital areas (Fig. 7*B*). In contrast, SpW with long-interval multiple spikes originated in central parietal and occipital areas. The pattern of propagation of the first and second spikes in any given SpW was different: the first spike propagated slowly to left temporal and frontal regions, whereas the second spike did not (Fig. 7*C*). Maximal propagation time for the first spike in these vents (earliest to latest detected spike across cortex) was 98 ± 12 ms. Overlay of the occurrence of both spikes in each SpW demonstrated that only central parietal and occipital regions displayed multiple long-interval spikes per SpW. In the case of SpW with short-interval multiple spikes, the origins of the first and second spikes were clearly different (Fig. 7*D*). The first spike in a multispike SpW originated in left lateral areas (as for the single-spike SpW events), whereas the second spike occurred first under occipital sensors. Propagation of the first spike in each case was significantly more rapid than for the SpW events containing long-interval spikes (49 ± 7 vs. 98 ± 12 ms, *P* < 0.01, *n* = 5), and overlay of the spatial maps for each spike shows only left temporal and frontal areas demonstrated multiple spikes per SpW.

## DISCUSSION

The base model used to generate SpW in the present work generated frequencies of events similar to those seen clinically for atypical absences and some manifestations of focal epilepsies (Blumenfeld 2005; Takahashi et al. 2015). Building on this baseline of stereotyped, single spike per SpW discharges, we have shown that different pathologies linked to genetic risk factors (enhanced NMDA conductance, reduced GABA_A_ receptor-mediated synaptic inhibition) generated multiple spikes per SpW with different spatiotemporal dynamic properties.

In all cases, the wave component of each SpW event correlated with an intense, long-lasting burst event in L5 IB neurons in both experimental and computational models (Figs. 1 and 2). Cross-correlating L5 IB membrane potential with concurrent L5 field potential recordings revealed that >90% of the correlation derived from the wave component of the field, with poor correlation values for the high-pass filtered spike component (Fig. 2*E*). These events represented a more intense version of the repetitive bursting seen in delta rhythms (Carracedo et al. 2013; Keller et al. 2010; Sanchez-Vives and McCormick 2000; Ulbert et al. 2004). In the case of single spikes per SpW and multiple long- or short-latency spikes, no significant change in this L5 behavior was seen (Fig. 4*A*). This supported the notion that the driving force behind the wave component of each SpW was essentially an excessive, but otherwise normal, component of neocortical network function (Blumenfeld 2005).

Enhancing excitation from deep layers up to superficial layers generated multiple superficial layer bursts caused by burst activity in L5 RS neurons. In this respect, the local circuit mechanism appeared similar to the theta-frequency interlaminar interactions observed nested within normal delta rhythms (Carracedo et al. 2013), but with intense L5 RS bursts altering the balance of interlaminar interactions in favor of this backward, ascending pathway. This pathway is weak in terms of connected pairs (Lefort et al. 2009) but is highly plastic, utilizing a large proportion of NMDA receptors in late EPSP generation on repeated activation. This has been shown for distal dendritic inputs onto L5 neurons (Cauller and Connors 1994) and also onto L2/3 neurons (Holmgren et al. 2003). The initial activation of L5 RS neurons via inputs from superficial layers appeared to be drowned out by a combination of the switch from single L5 RS spiking to burst spiking (Fig. 3) and the enhanced efficacy of the backward-propagating excitatory input from L5 to L2/3 RS pyramids. Previous experiments have shown that the second theta-frequency event nested within a delta period is already generated by ascending inputs from L5 in physiological conditions (Carracedo et al. 2013). Thus the reciprocal interlaminar interaction seen during normal delta rhythms was replaced by an entirely backward-propagating interaction (cf. Fig. 5, *Di* and *Diii*). Such a derangement of interlaminar interactions would be expected to interfere with the competition for cortical space suggested to underlie normal cortical function during cognition (Adesnik and Scanziani 2010).

The above-described pattern of SpW with long-latency (theta frequency) multiple field spikes did not resemble the decades-old “low-magnesium” model of seizures, which causes blanket elevation on NR2A/B-containing NMDA receptors (Hegstad et al. 1989). This was despite a global gain of function mutation in NR2A (present in all neocortical layers (Tongiorgi et al. 2003) being associated with absences in patients (Carvill et al. 2013). This may reflect the absence of an underlying delta rhythm in previous models of low-magnesium seizures or, more likely, a specific role for NR2A-containing NMDA receptors in modulating the strength of the backward-propagating pathway.

Reducing superficial layer inhibition generated multiple superficial layer bursts at shorter interburst intervals compared with the TBS model discussed above. In this case the dynamics of the field spikes seen on each SpW pointed to different generator mechanisms. First, the shorter interspike interval seen in the experimental model, patients, and patient tissue could be replicated entirely independently of deeper layers (Fig. 5*Cii*). In this case trains of bursts of action potentials were seen at low-beta frequency, a phenomenon also evident in the patient recordings taken using EEG (*patient 1*) and in temporal lobe tissue postresection (*patient 3*; see Fig. 6), suggesting a potential disconnect between the generation of these faster field spikes in superficial layers and the underlying large wave events coming from deeper layers. Second, Granger scores for SpW with multiple short-interval spikes at beta frequency suggested that all the interlaminar interactions in each SpW were in the forward-projecting direction (superficial layers to deep layers; Fig. 5*Div*).

Mutations in γ2-subunits of the GABA_A_ receptor are associated with absence epilepsies (Wallace et al. 2001). In mouse models, mutations generate very large delta-frequency epileptiform events, often with multiple, fast spikes superimposed (Kang et al. 2015). This subunit is expressed in both deep and superficial layers (Frola et al. 2013). In the deep layers, deficits in GABA_A_ receptor-mediated inhibition have been shown previously to generate very large delta rhythms (Carracedo et al. 2013), but the present data suggested that the fast, multiple field spikes per SpW arose from superficial layers. The use of peptidergic agents to reduce GABA release allowed focus predominantly on superficial layer interneurons (Lee et al. 2010). Reduced NPY-mediated signaling may have a direct impact on absence seizure severity, with the NPY agonist valproate being an effective form of therapy (Callaghan et al. 1982).

Taken together, these models suggest that different mechanisms, with different consequences for disrupting cortical dynamics, can generate SpW with different multiple field spikes, even in the same patient (Fig. 6). It should be noted that relative contributions from activity in different cortical layers could also alter the profile of SpW irrespective of the number and type of spikes (Fig. 5*C*). Thus the magnitude and polarity of spikes seen with noninvasive recordings may vary with EEG electrode location (Rappelsberger et al. 1982) in a manner depending on the size and orientation of the dipoles generated locally to the electrode (Tong and Thakor 2009). However, irrespective of cortical layer, the difference in the interspike interval when long- or short-interval multiple spikes per SpW were seen was invariant, suggesting that this measure of spike heterogeneity may be more robust than magnitude or polarity measures. In addition, there were clear differences in the propagation speeds of SpW containing long- and short-interval multiple spikes across the cortical mantle in the single patient’s data, shown as an exemplar (Fig. 7). The computational and experimental models used in this work predicted that short-interval SpW were generated by disinhibition, whereas the long-interval SpW were generated by excessive interlaminar excitation. Disinhibition has been shown to increase the velocity of epileptiform wave fronts (Hall and Kuhlmann 2013; Trevelyan et al. 2007). Thus the relative differences in propagation of these two SpW subtypes in the patient studied also add weight to the suggestion that SpW heterogeneity could possibly be used in the clinical setting to inform on the nature of the underlying pathology.

This heterogeneity of pathology corresponding to long- and short-interval multiple spikes may, in part, be responsible for the variety of symptoms associated with electrographic seizures containing SpW, mainly poor memory performance, reduced attention, and language deficits (van Rijckevorsel 2006). These symptoms can be severe (Caplan et al. 2008), but the severity and extent vary hugely from patient to patient. This may be related not just to the severity (in terms of number and duration of seizures) and extent of the epileptiform activity across the cortical mantle but also to the relative degree and type of interlaminar communication disruption seen (see above). Delta rhythm generation during wakefulness is closely associated with decision making (Nácher et al. 2013). Similarly, theta rhythms (the frequency of the long-interval field spikes modeled in the present study) are also strongly associated with recognition memory and are thought to be vital for processing speech (Doelling et al. 2014). Dynamic interactions between these two spectral bands are vital for normal cognitive function (Canolty and Knight 2010). A framework for the physiological processes underlying this interaction was set out in Carracedo et al. (2013), but the degree of disruption caused by the three different types of SpW seen in the present study needs further examination to directly relate to absence-associated cognitive deficits.

In addition, both delta and the shorter latency field spikes in SpW seen in the disinhibition model (ca. low-beta frequency) are closely associated with motor control (Hall et al. 2014; Jenkinson and Brown 2011) and cognitive function, with excessive beta-frequency activity related to deficits in motor performance and flexibility in cognitive control (Engel and Fries 2010). Thus the relative manifestations of long- and short-interval spikes in SpW may perhaps relate to the variability in some of the symptoms associated with absence epilepsies. Our present data suggest that the cortical dynamic landscape during absence seizures can be disrupted in a highly variable manner, even in simple animal models. These findings suggest this variability manifests both in the frequency of epileptiform events in superficial layers of neocortex and in the degree of interlaminar communication deficits in different cortical regions. Thus, far from being a highly stereotyped form of epilepsy, absence seizures demonstrate a complex profile of electrographic signatures and underlying pathologies.

## GRANTS

We thank The Wellcome Trust and The Wolfson Society for funding this work in the UK. R. D. Traub was funded by IBM and National Institute of Neurological Disorders and Stroke Grant R01NS044133.

## DISCLOSURES

No conflicts of interest, financial or otherwise, are declared by the authors.

## AUTHOR CONTRIBUTIONS

S.P.H., R.D.T., N.E.A., and M.A.W. conceived and designed research; S.P.H., N.E.A., M.O.C., I.S., A.J.J., and M.A.W. performed experiments; S.P.H., R.D.T., N.E.A., I.S., and M.A.W. analyzed data; R.D.T., N.E.A., and M.A.W. interpreted results of experiments; S.P.H., R.D.T., N.E.A., and M.A.W. prepared figures; S.P.H., R.D.T., and M.A.W. drafted manuscript; R.D.T. and M.A.W. edited and revised manuscript; S.P.H., R.D.T., N.E.A., M.O.C., I.S., A.J.J., and M.A.W. approved final version of manuscript.
